# Mechanisms of Kaposi's Sarcoma-Associated Herpesvirus Latency and Reactivation

**DOI:** 10.1155/2011/193860

**Published:** 2011-05-09

**Authors:** Fengchun Ye, Xiufen Lei, Shou-Jiang Gao

**Affiliations:** ^1^Tumor Virology Program, Greehey Children's Cancer Research Institute, The University of Texas Health Science Center at San Antonio, San Antonio, TX 78229, USA; ^2^Department of Pediatrics, The University of Texas Health Science Center at San Antonio, San Antonio, TX 78229, USA; ^3^Cancer Therapy and Research Center, The University of Texas Health Science Center at San Antonio, San Antonio, TX 78229, USA

## Abstract

The life cycle of Kaposi's sarcoma-associated herpesvirus (KSHV) consists of latent and lytic replication phases. During latent infection, only a limited number of KSHV genes are expressed. However, this phase of replication is essential for persistent infection, evasion of host immune response, and induction of KSHV-related malignancies. KSHV reactivation from latency produces a wide range of viral products and infectious virions. The resulting *de novo* infection and viral lytic products modulate diverse cellular pathways and stromal microenvironment, which promote the development of Kaposi's sarcoma (KS). The mechanisms controlling KSHV latency and reactivation are complex, involving both viral and host factors, and are modulated by diverse environmental factors. Here, we review the cellular and molecular basis of KSHV latency and reactivation with a focus on the most recent advancements in the field.

## 1. Introduction

Kaposi's sarcoma-associated herpesvirus (KSHV) was identified in an acquired immune deficiency syndrome (AIDS) patient with Kaposi's sarcoma (KS) [1]. Extensive studies have shown that KSHV is etiologically associated with KS, a vascular malignancy of endothelial cell origin, mostly involving the skin, oral cavity, and/or other subcutaneous tissues [2]. Clinical features of KS lesions include proliferation of KSHV latent nuclear antigen- (LANA- or LNA-) positive spindle-shaped tumor cells, extensive “slit-like” vascular networks, and infiltration of various inflammatory cells and red blood cells [3]. There are four clinical forms of KS: (1) classical KS, which is mainly seen in elderly men of Mediterranean and Eastern European origins, (2) endemic KS in Africa, (3) epidemic AIDS-related KS (AIDS-KS), and (4) iatrogenic KS in patients undergoing organ transplantation-related immunosuppression regimens. In Western countries, AIDS-KS is the most prevalent form of KS, which is also the most common malignancy in HIV patients [3]. KSHV is etiologically associated with all forms of KS. In addition to KS, KSHV is also causally implicated in several non-Hodgkin lymphomas including primary effusion lymphoma (PEL) and multicentric Castleman's disease (MCD) [4–6]. 

Like all herpesviruses, the life cycle of KSHV consists of latent and lytic replication phases [7]. In immunocompetent individuals, KSHV establishes latent infection following an acute infection. During latent infection, KSHV genome persists as a circular double-stranded DNA molecule (episome) in the nucleus with most viral genes being silenced except a few viral latent genes located in the latency locus. As a result, there is no production of virions. Latent infection allows KSHV to evade the host immune surveillance and facilitate the establishment of a lifelong persistent infection. KSHV latent cells constitute a reservoir of chronic viral infection tightly controlled by the host immune system. Latent infection has an essential role in the development of KSHV-associated malignancies because most tumor cells in KS, PEL, and MCD are latently infected by KSHV. 

In immunocompromised hosts, KSHV latent cells can be reactivated into lytic replication expressing all viral lytic genes and producing infectious virions. One of the first lytic genes to be expressed is an immediate early (IE) gene RTA (ORF50), followed by early genes such as MTA (ORF57) and K-bZIP (ORF-K8), and late genes such as major capsid protein ORF25. Viral DNA replication, capsid packaging, and virion maturation and egress also follow the expression of viral lytic genes, leading to the completion of viral lytic replication cycle. Lytic DNA replication generates a linear form of double-stranded DNA molecules, one copy of which is packaged into each virion. For herpesviruses, lytic replication not only produces infectious virions for spreading but also often causes their associated diseases. For KSHV, viral lytic products and *de novo* infection promotes cell proliferation, angiogenesis, and local inflammation, leading to the initiation and progression of KS tumors [8–19]. The importance of KSHV lytic replication for supporting KS tumors is substantiated by clinical observation that KS progression is tightly correlated with KSHV lytic antibody titers and viral loads in patients [20–27]. In KS tumors, a small subset of cells also undergoes spontaneous lytic replication. Inhibition of KSHV lytic replication with antiherpesviral drugs that block lytic replication causes KS tumor regression [28–30]. 

Since both latent and lytic replication phases are important for the development of KS tumor, understanding the mechanisms of KSHV latency and reactivation might hold the key to elucidating KSHV-induced pathogenesis, as well as developing novel therapeutic approaches, and thus has been the hot topic in the field. Here we attempt to review the most recent advancements in the molecular and cellular mechanisms that regulate KSHV life cycle.

## 2. Mechanism of KSHV Latency

A successful KSHV latency program must ensure (1) silencing of viral lytic gene expression; (2) survival and continuous proliferation of the infected cells; (3) stable maintenance, replication, and proper segregation of the viral episomes into daughter cells during mitosis. Although how these criteria are achieved is still not fully understood, it is clear that establishment of viral latency is mediated by complex host-virus interactions involving both the host defense systems and the viral surviving capacities [31]. 

### 2.1. Host Factors

The host immune surveillance plays a key role in eliminating the virus during an acute infection and keeping the virus in check throughout the lifelong persistent infection. However, it is not known whether host immune responses directly suppress KSHV lytic replication. At the cellular level, several studies have demonstrated that KSHV acute infection elicits antiviral defenses, which inhibit viral gene expression and lytic replication. Attachment of KSHV virions to the target cells triggers a rapid secretion of interferon-beta (IFN-*β*) and activates interferon regulatory factor 3 (IRF-3) to induce the expression of host IFN-stimulated antiviral genes [32]. KSHV glycoprotein K8.1 appears to trigger this event. In addition, interferon-alpha (IFN-*α*) blocks the initiation of lytic replication while interferon-gamma (IFN-*γ*) inhibits the late steps of lytic replication [33–38]. Furthermore, cellular interferon regulatory factor 7 (IRF-7) negatively regulates lytic replication by competing with the lytic switch protein RTA for binding to RTA-responsive element (RRE) in the MTA promoter [39, 40]. IRF-7 participates in IFN-*α* repression of RTA-mediated transactivation [39]. The inhibition of lytic gene expression and replication by antiviral responses promotes viral latency and survival of the infected cells. Thus, KSHV has evolved to adapt to the host innate antiviral responses to achieve infection and establish latency in the host. In the same time, KSHV promotes latency by modulating multiple cellular pathways to favor cell growth and survival, which will be discussed in the latter section.

### 2.2. Viral Genome and Viral Genes

During latency, KSHV persists as circular episomes associated with histones in the nucleus [41]. These episomes are assembled into nucleosomal structures that resemble bulk cellular chromatin. All viral lytic genes are near complete shut down during latency. When switched into lytic replication, the chromatins of KSHV genomes undergo significant modifications [42, 43]. Two recent studies have examined the epigenetic landscape of KSHV genomes using high-resolution tiling microarrays in conjunction with immunoprecipitation of methylated DNA (MeDIP) or modified histones (chromatin IP, CHIP) [44, 45]. During *de novo* infection of endothelial cells, KSHV genomes undergo heavy methylation at CpG dinucleotides [44]. These alterations of DNA methylation patterns are accompanied by specific histone modifications leading to the rapid establishment of latency. Interestingly, viral genomes are not uniformly modified. In particular, activating histone modification marks such as H3K9/K14-ac and H3K4-me3 persist at several loci including the RTA promoter [44]. Similarly, in latent BCBL-1 cells, KSHV genomes contain both activating marks H3K9/K14-ac and H3K4-me3 as well as repressive marks H3K9-me3 and H3K27-me3, which are mutually exclusive on most part of the latent genome [45]. Both activating mark H3K4-me3 and repressive mark H3K27-me3 are also present in region encoding RTA and ORF48, which change upon viral reactivation. These findings obviously have generated new questions, one of which is how viral lytic regions are suppressed during viral latency? Intriguingly, rapid and widespread deposition of H3K27-me3 is present across the entire viral genomes during *de novo* infection [44]. These bivalent modifications are also present in latent cells [45]. Because this bivalent modification is known to repress transcription in spite of simultaneous presence of activating marks, it is postulated that these modification patterns could induce a poised state of repression during latency that could be rapidly reversed once the lytic cycle is triggered. Interestingly, bivalent modification mark H3K27-me3 is colocalized with EZH2, an H3K27-me3 histone methyltransferase of the polycomb group proteins (PcG) [45]. During viral reactivation, EZH2 is dissociated from the regions encoding for IE genes, accompanying the decrease of H3K27-me3 and the increase of transcriptional activities. 

Together, results of these studies suggest that epigenetic modifications of KSHV genome through histone modifications are essential in silencing lytic gene expression and promoting viral latency [44, 45]. Interestingly, there is a substantial reduction of repressive mark H3K27-me3 in the latency locus compared to the rest of KSHV genome during latency. This region encodes LANA, vCyclin (ORF72), vFLIP (ORF71), and a cluster of 25 matured microRNAs (miRs) derived from 12 precursor miRs (miR-K1-12) [46, 47]. LANA, vCyclin, and vFLIP are transcribed as multiple polycistronic mRNAs, including a 5.8 kb unspliced and a differentially spliced 5.3 kb mRNAs, both of which encode all three ORFs, and another 2.8 kb mRNA, which only encodes vCyclin and vFLIP [46]. Consistent with results of the epigenetic studies, these proteins, along with the miRs, are the only viral genes expressed in latently infected KS cells. They, along with LANA-2/vIRF3 (ORF-K10.5), are also the only ones expressed in latently infected PEL cells. It is suggested that the expression of latent genes in the latency locus might also be controlled at the chromatin level [48]. Cohesins and the 11-zinc finger protein CCCTC-binding factor (CTCF), which mediate sister chromatin cohesion and functions at chromatin boundaries, respectively, play key roles in the structural and functional organization of chromosomes [49]. Both cohesion and CTCF bind to KSHV episomes and have specific binding sites located within the control region of the major latency transcript that encodes LANA, vCyclin, cFLIP, and miRs. Disruption of the interactions between these *trans* and *cis *elements not only results in failure in colony formation in 293 cells but also leads to enhanced expression of adjacent KSHV lytic genes such as vOX (ORF14) and vGPCR (ORF-K14). Interestingly, KSHV genes regulated by CTCF cohesin are under cell cycle control and that mutation of the CTCF binding sites abolished cell cycle-regulated transcription [48]. Cohesin subunits assembled at the CTCF binding sites and bound CTCF proteins in a cell cycle-dependent manner. Subcellular distribution of CTCF and colocalization with cohesins also varied across the cell cycle. RAD21 and SMC1 are associated with the cellular CTCF sites at mammalian *c-myc* promoter and H19/Igf2 imprinting control region. It is postulated that this latency control region is occupied with the cellular chromatin boundary factor CTCF and chromosome structural maintenance proteins SMC1, SMC3, and RAD21, which comprise the cohesin complex. Indeed, RAD21 is rapidly dissociated from the viral genome during reactivation. Knockdown of RAD21 results in a transcriptional upregulation of the lytic genes vOX and vGPCR, while overexpression of RAD21 represses transcription of CTCF targeted lytic genes. Furthermore, a RAD21-CTCF chimeric protein converts CTCF into a transcriptional repressor of KSHV genes that are normally activated in the G2 phase of cell cycle. Thus, CTCF-cohesin complex plays a critical role in regulating cell cycle control of viral gene expression during latency, and failure to maintain cell cycle control of latent transcripts inhibits host cell proliferation and survival.

Because LANA, vCyclin, vFLIP, and the miRs are located in the latency locus and expressed during latency, they are likely to contribute to KSHV latency. Extensive studies have indeed indicated that all of these genes play important roles in suppressing KSHV lytic gene expression and promoting latency. Furthermore, they are also involved in KSHV-induced malignant transformation by promoting cell growth and survival. 

#### 2.2.1. LANA

LANA encoded by ORF73, also known as latent nuclear antigen (LNA), was initially identified as a doublet of 220–230 kDa nuclear proteins by Western blotting, and as the most immunodominant latent antigen in KS patients [50]. LANA is strongly expressed in KSHV-infected cells throughout the virus life cycle [50–54]. LANA is responsible for KSHV episomal DNA replication, maintenance, and segregation into daughter cells during mitosis [55–58]. The terminal repeats (TRs) in KSHV genome contain a DNA replication origin *cis*-element, and LANA is a *trans*-acting protein that preferentially binds to TRs to form a DNA-protein complex [59–61]. In lymphoblasts, LANA is necessary and sufficient for the persistence of an artificial episome containing two TR units [62–64]. Immunostaining reveals that LANA is colocalized with the artificial episomes in nuclei and along mitotic chromosomes, supporting a model in which LANA tethers KSHV DNA to chromosomes during mitosis to enable the efficient segregation of KSHV episomes to progeny cells [60]. Both C- and N-terminals of LANA mediate its binding to TRs [59, 65–67]. Despite the absence of a DNA polymerase activity by LANA, the LANA-TRs complex is sufficient for episomal DNA replication [62], indicating that the complex may recruit cellular DNA replication machinery to complete viral DNA replication. Indeed, poly(ADP-ribose) polymerase 1 (PARP1) and other known replication factors such as origin recognition complex 2 (ORC2), CDC6, uracil DNA glycosylase 2 (UNG2), and Mcm7 bind to TRs [68–73]. The association of TRs with ORCs is LANA dependent, and both the N- and C-terminals of LANA are required for this interaction [65, 70, 74, 75]. Thus, LANA's role in KSHV episomal DNA replication is mediated through its binding to TRs and recruitment of cellular DNA replication proteins.

LANA also tethers viral episomes to host chromosomes during mitosis, ensuring proper partitioning of viral episomes into daughter cells [55]. Fine molecular mapping identified amino acids 5 to 22 of LANA as the region that binds to chromosomes [74–77]. Both methyl-CpG-binding protein MeCP2 and the 43-kDa protein DEK interact with LANA and might mediate the tethering [78, 79]. However, LANA also interacts with bromodomain protein Brd4 through the extra-terminal domain of Brd4 and a carboxyl-terminal region of LANA [80]. Since Brd4 is associated with mitotic chromosomes throughout mitosis and is colocalized with LANA and KSHV episomes on host mitotic chromosomes, Brd4 might also be an interacting protein that mediates LANA tethering on mitotic chromosomes. Similarly, LANA is associated with the nuclear mitotic apparatus protein (NuMA) [58]. In synchronized cells, NuMA and LANA are colocalized in interphase cells. During mitosis, the two proteins are separated from each other at the beginning of prophase but are reassociated at the end of telophase and cytokinesis. Silencing of NuMA expression by siRNAs disrupts the association of NuMA with microtubules resulting in the loss of KSHV TR plasmids containing the latent origin of replication. A recent study showed that LANA is associated with centromeric protein F (CENP-F) as speckles, some of which are paired at centromeric regions of a subset of chromosomes in KSHV-infected JSC-1 cells [81]. Moreover, LANA is associated with kinetochore protein Bub1 (budding uninhibited by benzimidazole 1) known to form a complex with CENP-F [81]. The dynamic association of LANA and Bub1/CENP-F and the colocalization among Bub1, LANA, and the KSHV episome tethered to the host chromosome were shown in fluorescence in situ hybridization (FISH). Knockdown of Bub1 expression by lentivirus-delivered shRNA dramatically reduced the number of KSHV genome copies, whereas no dramatic effect was seen with CENP-F knockdown. Therefore, the interaction between LANA and the kinetochore proteins CENP-F and Bub1 is important for KSHV genome tethering and its segregation to new daughter cells, and Bub1 potentially plays a critical role for the long-term persistence of the viral genome in the infected cells. 

Collectively, these results indicate that LANA is essential in KSHV episomal DNA replication, maintenance, and segregation of the viral genomes into daughter cells during mitosis. Consistent with these results, genetic disruption of LANA led to rapid loss of KSHV genomes in human cells and prevented KSHV from establishing persistent infection [82].

Another important mechanism in which LANA promotes KSHV latency is through inhibition of viral lytic gene expression. Although LANA positively regulates the expression of some genes, it also interacts with mSin3 corepressor complex [83] and a nuclear transcriptional repressive protein named “KLIP-1” (KSHV LANA-interacting protein 1) to exert a negative regulatory effect on gene transcription [84]. Consistent with these observations, LANA induces and relocates RING3 to nuclear heterochromatin regions, creating a local euchromatic microenvironment around the viral episomes that are anchored to the heterochromatin [85, 86]. In addition, LANA interacts and recruits DNA methyltransferases (Dnmts) to repress gene expression through chromatin methylation at the promoter regions [87]. Indeed, LANA inhibits the expression of RTA at the transcriptional level [88]. There is evidence that LANA is recruited to RTA promoter and participates in local chromatin remodeling to suppress its expression [43]. Interestingly, LANA also directly interacts with RTA and inhibit its transactivation activity [89]. LANA physically associates with recombinant signal sequence-binding protein-J kappa (RBP-J*κ*) *in vitro* and in KSHV-infected cells forming complex on the RBP-Jkappa cognate sequences [90]. Of note, the RBP-J*κ* binding sites within the RTA promoter are critical for LANA-mediated repression. Thus, LANA maintains KSHV latency by targeting a major downstream effector of the Notch signaling pathway [90]. In agreement with these data, deletion of LANA increased the expression levels of all classes of lytic genes including RTA, MTA, vIL-6 (ORF-K2), ORF59, and ORF-K8.1 with and without induction with 12-*O*-tetradecanoyl-phorbol-13-acetate (TPA) and sodium butyrate [91]. This enhancement of viral lytic gene expression was also observed following overexpression of RTA with or without simultaneous chemical induction. LANA mutant cells produced more infectious virions than the wild-type virus cells did. Furthermore, genetic repair of the mutant virus reverted the phenotypes to those of wild-type virus. Together, these results demonstrated that, in the context of viral genome, LANA contributes to KSHV latency by inhibiting the expression of RTA as well as inhibiting its transactivation of downstream genes. Nevertheless, the expression of RTA and RTA's transactivation in the LANA mutant can be further increased by treatment with TPA and butyrate, indicating that KSHV latency is controlled by multiple mechanisms [91]. 

LANA also contributes to latency by promoting cell growth and survival. LANA interacts with and functionally inhibits tumor suppressors p53 and pRb to promote growth and survival of KSHV-infected cells [92–95]. Nevertheless, LANA inhibition of p53 function is not complete at least in PEL cells as these cells respond quickly to DNA damaging agents. In addition, LANA stabilizes *β*-catenin by binding to the negative regulator GSK-3*β*, causing a cell cycle-dependent nuclear accumulation of GSK-3*β* [96, 97]. Furthermore, LANA stimulates cell cycle progression by interacting with, stabilizing, and activating oncogene c-Myc [98]. Interestingly, siRNA knockdown of the expression of c-Myc in PEL cells caused virus reactivation and increased the expression of lytic genes in addition to inhibition of proliferation and increase of apoptosis [99]. c-Myc overexpression inhibited lytic gene expression and virus reactivation. These results suggest that c-Myc is required for maintenance of KSHV latency through suppression of lytic gene expression and promotion of cell survival and proliferation and that LANA contributes to KSHV latency by stabilizing and activating c-Myc.

#### 2.2.2. vCyclin

vCyclin forms an active kinase complex with cellular CDK6 to regulate cell cycle progression by phosphorylating pRb protein, a common substrate of the cellular cyclin D-CDK6 complex, as well as a large repertoire of unique cellular substrates such as p27KIP1, p21CIP1, ORC1, and CDC6 involved in cell cycle regulation [100–104]. Thus, vCyclin might contribute to KSHV latency by promoting cellular proliferation. The vCyclin-CDK6 complex also phosphorylates nucleophosmin (NPM), facilitating NPM-LANA interaction and recruitment of HDAC1 to regulate KSHV latency [105]. Nevertheless, the role of vCyclin in regulating KSHV life cycle remains largely unclear. Further study in the context of virus infection by reverse genetics might shed light on its function. 

Interestingly, murine gammaherpesvirus 68 (MHV68), which is closely related to KSHV, also encodes a cyclin homolog. Deletion of vCyclin has little effect on MHV68 growth kinetics and viral titer *in vitro *[106]. However, the mutant virus fails to reactivate for lytic replication *in vivo *following acute infection in mice. The growth deficiency of the mutant virus is not mediated by T or B cells because it is present in both immune competent BALB/c mice and T- and B-cells deficient SCID mice [106]. These results indicate that MHV68 vCyclin might mediate important functions in the acute infection and is required for efficient reactivation from latency. Given the close phylogenic relationship between KSHV and MHV68, it is possible that KSHV vCyclin might have similar functions.

#### 2.2.3. vFLIP

vFLIP, like its cellular counterpart cFLIP, inhibits Fas ligands-induced apoptosis by inhibiting procaspase-8 activation [107]. In addition, vFLIP activates both the canonical and noncanonical NF-*κ*B pathways to promote cell survival [108–111]. Thus, vFLIP likely contributes to KSHV latency by promoting cell survival. While the effect of NF-*κ*B pathway on KSHV lytic replication is controversial and appears to be context dependent [112–115], genetic deletion of vFLIP from the KSHV genome showed that it inhibits the expression of RTA and suppresses KSHV lytic replication [116]. Mechanistically, vFLIP suppresses viral gene expression by inhibiting AP-1 pathway through activation of NF-*κ*B pathway. Thus, vFLIP regulates KSHV latency by activating a key cellular survival pathway to promote cell survival and inhibiting viral lytic replication.

#### 2.2.4. MicroRNAs

Among the 12 pre-miRs encoded by KSHV, ten (miR-K1–9 and -K11) form a cluster in an intron between vFLIP and kaposin, while the remaining two reside in the coding region (miR-K10) and 3′UTR of ORF-K12 (miR-K12), respectively [117–119]. All KSHV miRs are expressed during latency, but the expression of miR-K10 and -K12 is further increased upon lytic induction [117–119], suggesting their potential roles in viral latency. Indeed, genetic deletion of the miR cluster inhibits the expression of KSHV lytic genes in both uninduced cells and cells induced for lytic induction [120, 121]. Among these miRs, miR-K9 directly targets RTA to regulate viral replication [122]. Several KSHV miRs also regulate viral life cycle through indirect mechanisms. miR-K5, -K9, and -K10a/b target Bcl-2-associated factor (BCLAF1) to inhibit apoptosis and regulate lytic replication [123], while miR-K4-5p epigenetically regulates lytic replication by targeting Rb-like protein 2 (Rbl2) to increase the expression of DNMT1, -3a and -3b [124]. miR-K1 targets I*κ*B*α* to increase cellular NF-*κ*B activity [120]. Expression of miR-K1 in mutant cells rescues the NF-*κ*B activity and inhibits viral lytic replication, while suppression of miR-K1 in KSHV-infected BCP-1 cells increases the I*κ*B*α* protein level, suppresses NF-*κ*B activity, and increases the expression of viral lytic genes [120]. Because NF-*κ*B pathway is involved in cell growth and survival, immunity, inflammation, and tumor development, similar to vFLIP, miR-K1 might directly contribute to the pathogenesis of KSHV-induced malignancies in addition to its function in regulating viral life cycle. miR-K3 suppresses viral lytic replication by targeting nuclear factor I/B, which is shown to activate the RTA promoter [121]. miR-K11 is an ortholog of human miR-155 [125, 126] that targets BACH1. Consequently, several BACH1-regulated genes including heme-oxygenase-1 (HMOX-1) and xCT are upregulated by miR-K11 [127]. HMOX-1 enhances cell survival and proliferation, while xCT replenishes intracellular glutathione stores to maintain cell viability in an environment of oxidative stress. Thus, by targeting the transcriptional repressor BACH1, miR-K11 promotes host cell survival, particularly in an oxidative stress environment. Finally, KSHV miR-K10a targets TNF-like weak inducer of apoptosis (TWEAK) receptor (TWEAKR) [128]. Downregulation of TWEAKR by miR-K10a results in reduced levels of TWEAK-induced caspase activation and apoptosis as well as decrease in expression of proinflammatory cytokines IL-8 and MCP-1 in response to TWEAK. Thus, miR-K10a protects cells from apoptosis and suppresses proinflammatory responses, which might contribute to KSHV latency [128].

#### 2.2.5. Antisense RNAs

Antisense transcripts are widespread in mammalian cells [129]. Global transcriptome analyses show that up to 70% of transcripts have antisense partners and that perturbation of antisense RNAs can alter the expression of the sense genes. The biological functions of these antisense RNAs are not fully understood. Analyses of KSHV-infected cells by deep sequencing and genome-wide tiling array revealed extensive antisense transcription during lytic replication throughout the KSHV genome, including a 10 kb RNA antisense to the major latency locus [130, 131]. It is postulated that these antisense RNAs might play a role in KSHV life cycle by regulating viral chromatins. Interestingly, an antisense transcript to RTA mRNA does not regulate RTA expression but encoding small peptides with unknown functions [132]. 

**Figure 1:**
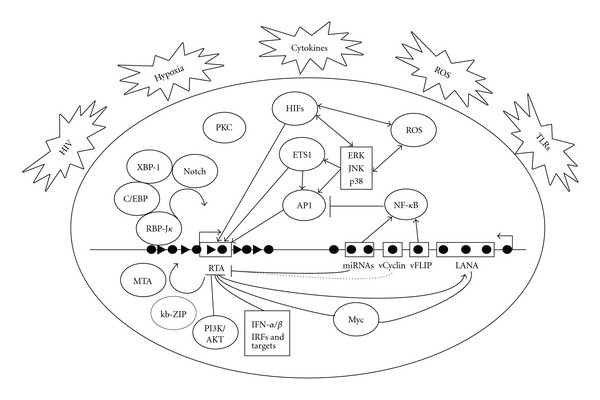
Schematic illustration of mechanisms of KSHV latency and reactivation. During primary infection, KSHV activates innate immune responses such as IFN-*α*/*β* and IRFs and multiple cell survival pathways including NF-*κ*B, c-Myc, and PI3K/AKT, all of which repress KSHV lytic replication. Expression of KSHV latent products including LANA, vCyclin, vFLIP, and a cluster of miRNAs also inhibits KSHV lytic replication. As a result, KSHV establishes latency following primary infection. During latency, most parts of viral genome are epigenetically silenced and contain both active and repressive histone marks (black triangle) with the exception of the latent locus, which is transcribed and contains only active histone marks (black cycle). KSHV latent products enhance/maintain latency by promoting cell survival and facilitating the viral episome replication and segregation. Several physiological factors including hypoxia, HIV infection, inflammatory cytokines, oxidative stress, and ROS can induce RTA expression by activating specific cellular pathways and transcriptional factors including MEK/ERK, JNK, p38, AP-1, Ets-1, HIF1/2, PKC, and Notch. RTA interacts with several host proteins such as XBP-1, Notch, and C/EBP*α*, as well as viral proteins such as MTA and kb-ZIP, to quickly remove the repression histone marks on KSHV genome, resulting in the expression of viral lytic genes and activation of the entire viral lytic transcriptional program.

In summary, each of the KSHV latent genes/products contributes to KSHV latency by playing a distinct but complementary role (Figure 1). Their functions are best illustrated by reverse genetic analyses showing that deletion of any one of these latent genes is not sufficient for maximum productive lytic replication albeit an enhanced viral lytic replication program. Thus, KSHV has evolved multiple synergistic mechanisms to cope with complex host and environmental factors so as to accomplish successful latency. 

## 3. Mechanism of KSHV Reactivation

### 3.1. Factors That Induce KSHV Reactivation from Latency

As discussed earlier, the immune system plays a key role in controlling KSHV life cycle. Indeed, productive lytic replication frequently occurs when the host suffers from a temporary or permanent immune suppression [3, 133]. Nevertheless, immune suppression may only provide a favorable physiological environment for the virus to thrive. While it might be necessary, it is likely insufficient to trigger KSHV switch from latency into lytic replication. Additional physiological or environmental factors are likely required to induce KSHV lytic replication. 

#### 3.1.1. Hypoxia

Hypoxia is suggested to be a possible cofactor for KSHV reactivation based on the clinical observation that KS tumors often appear on body parts such as feet and arms, where blood and oxygen supply might be relatively low compared to other body parts. In support of this hypothesis, it was demonstrated that hypoxia could induce KSHV lytic replication in PEL cells [134]. Hypoxia induces accumulation of hypoxia-inducible factors (HIF1/2). In KSHV genome, promoters of at least two lytic genes, RTA and ORF34, contain functional hypoxia response elements (HREs) [135, 136]. The RTA promoter mainly responds to HIF-2*α* while the ORF34 promoter responds to both HIF-1*α* and HIF-2*α* [135, 136]. In fact, the ORF34-37 promoter region has six consensus HREs, one of which (HRE2) plays a critical role in hypoxia induction of a 3.4 kb transcript encoding ORF35-37 and a 4.2 kb transcript encoding ORF34-37. Hypoxia also induces activation of transcription factor X-box binding protein 1 (XBP-1), which can synergistically transactivate RTA promoter with HIF-1*α* to induce KSHV lytic replication under hypoxia condition [137]. In addition, LANA switches from repressive to active role by interacting with HIF-1*α* to upregulate RTA expression under hypoxia condition [138]. 

#### 3.1.2. HIV and Other Infections

AIDS-KS is more aggressive than other forms of KS. HIV infection is suspected to contribute to KSHV reactivation. HIV-1 infection of PEL cell line BC-3 enhances KSHV lytic replication [139, 140], and HIV-1 transactivator Tat is sufficient to induce expression of RTA and KSHV lytic replication [141]. In addition, infection by other viruses such as herpes simplex virus-1, human herpesvirus 6 (HHV6), or cytomegalovirus (CMV) may cause KSHV reactivation [142–144]. Toll-like receptors 7 and 8 (TLR7/8), which are activated by some viral infections, also mediate KSHV reactivation [145].

#### 3.1.3. Inflammation and Inflammatory Cytokines

Reactivation of KSHV by HIV and other viruses could be indirectly mediated by inflammation cytokines. While CD4^+^ T-cell counts are often low in HIV-infected patients, phagocytes and other types of lymphocytes continue to elicit inflammatory responses, resulting in production of various inflammatory cytokines. Some inflammatory cytokines that are highly expressed in KS lesions can reactivate KSHV [146]. While IFN-*γ* induces lytic replication in cultured BCBL-1 cells [34, 147], IFN-*α* inhibits lytic replication, and other cytokines such as IL-1, IL-2, IL-6, TNF-*α*, bFGF, and granulocyte-macrophage colony stimulating factor (GM-CSF) have little effect [33]. In endothelial cells, inflammation cytokines inhibit spontaneous KSHV lytic gene expression [148]. Obviously, the roles of cytokines in KSHV life cycle and their mechanisms of regulation remain unclear and need to be further defined.

#### 3.1.4. Oxidative Stress and Reactive Oxygen Species (ROS)

Since inflammation and oxidative stress are features of KS tumors, we speculate that reactive oxygen species (ROS) produced from these two physiological events might mediate KSHV reactivation. Indeed, we have shown that ROS hydrogen peroxide (H_2_O_2_) is sufficient to induce lytic replication in KSHV-infected BCBL-1 cells by activating the MAPK pathways, which are attenuated by H_2_O_2_-specific antioxidants [149]. Interestingly, ROS induced by inhibition of NF-*κ*B pathway also triggers KSHV reactivation [150], which is consistent with the observation that NF-*κ*B pathway inhibits KSHV lytic replication by suppressing AP-1 pathway [116]. Importantly, induction of KSHV reactivation by hypoxia and proinflammatory cytokines is mediated by ROS [149]. H_2_O_2_ is expected to be abundant in all clinical forms of KS: AIDS-KS patients associated with immunosuppression and inflammation [151], classical KS patients associated with ageing [152], transplant KS patients associated with immunosuppression [153], and African KS patients associated with excess iron [154, 155]. Thus, ROS could be the common physiological cue that converges different KSHV reactivation signals. Low levels of ROS are known to promote proliferation, cell survival, angiogenesis, and inflammation through REDOX signaling. Indeed, treatment with antioxidant N-acetyl-L-cysteine (NAC) not only inhibits KSHV lytic replication *in vivo* but also slows the development of PEL in a mouse xenograft model [149]. These results indicate that REDOX signaling regulates KSHV lytic replication *in vivo*, and antioxidants and anti-inflammation drugs could be promising preventive and therapeutic agents for effectively targeting KSHV replication and KSHV-related malignancies [149]. Because many of these agents are widely available and affordable, their use is attractive, particularly in the African settings [149].

Additional factors might also contribute to KSHV reactivation. For instance, several studies suggested that specific cell cycle phases and cell differentiation might regulate KSHV reactivation [156, 157]. In addition to the synergistic effect with hypoxia condition, XBP-1 alone can effectively initiate KSHV reactivation by activating the RTA promoter [157, 158]. Of note, splicing of the XBP-1 mRNA, which specifically occurs during B-cell differentiation, is critical in triggering KSHV reactivation. Thus, KSHV reactivation may be integrated into host cell differentiation program. Furthermore, some environmental factors such as excessive exposure to high concentration of iron (Fe^++^) or certain chemicals or diets have been suggested to be the risk factors for KS and might induce KSHV lytic replication [159–162].

### 3.2. Cellular Signaling Pathways Involved in Regulation of KSHV Lytic Replication

While various host and environmental factors can trigger KSHV reactivation, most of them exert their effects indirectly by activating specific cellular signaling pathways. Intracellular calcium mobilization induces Ca^++^-dependent KSHV reactivation, and inhibition of calcineurin signaling blocks KSHV reactivation [163]. Protein kinase C delta (PKCdelta) is also required for KSHV lytic replication [164]. Treatment with PKCdelta inhibitors GF109203X and rottlerin inhibits TPA-induced KSHV lytic replication. Systematic screening has identified a number of additional cellular factors and their associated pathways that might mediate KSHV reactivation [165]. The Raf/MEK/ERK/Ets-1 pathway is one of the pathways mediating KSHV reactivation [165]. Indeed, the promoters of RTA, MTA and K-bZIP, and origins of lytic replication (OriLyts) contain functional DNA-binding sites for AP-1, which is downstream of the pathway, and are responsive to AP-1 activation [166–168]. In primary infection of primary human umbilical vascular endothelial cells (HUVEC), KSHV activates MEK/ERK, JNK, and p38 MAPK pathways, all of which activate AP-1, to facilitate its entry into target cells and productive lytic replication at the early acute stage of infection [169, 170]. Similarly, all three MAPK pathways mediate both spontaneous and TPA-induced KSHV reactivation in latent KSHV-infected cells [168, 171, 172]. These pathways activate and induce the expression of many transcription factors such as AP-1 and Ets-1. It is interesting that all three pathways are required for KSHV lytic replication, suggesting the close interactions of these pathways. Recently, Pim-1 and Pim-3 kinases are found to mediate KSHV lytic replication [173]. Of interest, Pim-1 and Pim-3 are induced in response to physiological and chemical inducers of KSHV lytic replication. Pim-1 and Pim-3 phosphorylate LANA on serine residues 205 and 206 and reverse its repressive effect on the transcription and transactivation function of RTA. Furthermore, Toll-like receptors (TLRs), which recognize pathogens and are vital for the host innate immune responses, also mediate KSHV reactivation [145]. Agonists of TLR7/8 and vesicular stomatitis virus (VSV) known to activate these receptors can reactivate KSHV from latency. These results indicate that secondary infections by other pathogens such as HIV, other viruses, and bacteria might trigger KSHV reactivation by activating specific cellular pathways. 

KSHV itself has evolved multiple mechanisms to manipulate cellular antiapoptotic and survival pathways, each of which might affect its life cycle. As indicated above, pathways that promote cell growth and survival usually favor KSHV latency. Suppression of these pathways in latent cells disrupts latency and reactivates KSHV. For example, the cell survival pathway NF-*κ*B, which is activated by vFLIP, miR-K1, and several other viral lytic genes, has inhibitory effect on KSHV lytic replication [112, 116, 120, 174]. In addition to AP-1, NF-*κ*B also antagonizes RBP-J*κ* to negatively regulate the expression and transactivation function of RTA [174]. In KSHV-infected latent cells, inhibition of NF-*κ*B pathway disrupts viral latency and activates viral lytic replication [112]. Nevertheless, as indicated above, the role of NF-*κ*B pathway in KSHV life cycle is context dependent [115]. It is likely that the balance of AP-1 and NF-*κ*B pathways determines the fate of virus replication status in a given cell type. If the NF-*κ*B pathway is robust and represses the AP-1 pathway, KSHV would remain in latency. However, if AP-1 is strongly activated by other factors and it can not be repressed by the NF-*κ*B pathway, KSHV would undergo lytic replication regardless of the status of NF-*κ*B pathway. This mechanism of regulation might explain the contradictory role of NF-*κ*B pathway in KSHV life cycle in different cell types. For example, KSHV immediately enters latency following primary infection in dermal microvascular endothelial cells (DMVECs) [175, 176]. In contrast, KSHV undergoes a productive lytic phase, expressing almost all the lytic genes and producing a high titer of infectious virions following primary infection of HUVEC [9, 177, 178]. Infection of both HUVEC and DMVEC activates the ERK pathway; however, infection of HUVEC also activates the JNK and p38 pathways, while infection of DMVEC robustly activates the NF-*κ*B pathway [19, 169, 170, 179]. Additional studies are required to further define the role of NF-*κ*B pathway in KSHV life cycle in different experimental systems. Consistent with this notion, a recent study showed that inhibition of the prosurvival PI3K-Akt pathway favors KSHV reactivation from latency [180]. Inhibition of the pathway with Akt inhibitor VIII reactivates KSHV from latency by increasing RTA expression and activity. Similar to KSHV, MHV-68 infection induces PI3K-dependent Akt activation, and lytic replication of MHV-68 is enhanced when PI3K-Akt pathway is inhibited with both chemical inhibitors and siRNA knockdown.

### 3.3. Viral Genome and Genes

Several studies have provided evidences of epigenetic control of KSHV reactivation. One study showed that the promoter of RTA is heavily methylated in latent genome, and TPA treatment not only causes demethylation of RTA promoter but also induces KSHV lytic replication [181]. In addition, the RTA promoter is highly responsive to sodium butyrate and trichostatin A (TSA), inhibitors of histone deacetylases [182]. These two inhibitors target classes I and II histone deacetylases. Recently, we have shown that inhibition of class III histone deacetylase SIRT1 also induces RTA expression and activates KSHV lytic replication [183]. 

As stated above, most regions of the latent KSHV genome contain both activating and repressive histone marks in a mutually exclusive manner as well as PcG, which rapidly change upon reactivation [44, 45]. Inhibition of the expression of PcG complex protein EZH2 or H3K27me3-specific histone demethylases with a small molecule inhibitor DZNep or RNAi knockdown induced KSHV lytic replication [45]. These data indicate that histone modifications control the temporal and sequential expression of the lytic gene cascade, and the PcG proteins play a critical role in controlling KSHV latency. The regulation of spatial and temporal association of PcG proteins with the KSHV genome might be crucial for KSHV life cycle. 

Several KSHV genes are essential for lytic replication. Most of them are expressed early (IE and early genes) during KSHV lytic replication and thus are essential for the initiation and completion of the viral lytic cycle. The most important and best studied IE and early genes are RTA, MTA, and K-bZIP. 

#### 3.3.1. RTA

Expression of RTA is both necessary and sufficient for KSHV reactivation [184–186]. Genetic deletion of RTA results in defective reactivation and lytic DNA replication [187]. RTA autoactivates its own promoter [188] and transactivates downstream lytic genes including vIL-6 [189, 190], polyadenylated nuclear RNA (Pan) [191], MTA [192, 193], K-bZIP [192, 193], vIRF1 (ORF-K9) [194], ORF-K1 [195], small viral capsid protein (ORF65), ORF56, SOX (ORF37), vOX, and ORF52 [189, 194, 196]. RTA binds to the promoters of its targets by direct DNA binding or protein-protein interactions. Several RTA-activated promoters contain RRE [197]. Selective amplification of bound sequences *in vitro* identified a number of RTA direct binding targets [198]. Chip-on-Chip genome-wide screening for RTA direct targets in KSHV-infected BCBL-1 cells identified a total of 19 putative RTA binding sites located in the promoters, introns, or exons of KSHV genes. These targets include ORF8, ORFK4.1, ORFK5, PAN, ORF16, ORF29, ORF45, RTA, K-bZIP, ORFK10.1, ORF59, ORF-K12, ORF71/72, vOX/vGPCR (ORF74), ORF-K15, the two OriLyts, and the miR cluster [199]. Many of these targets have been experimentally confirmed or identified in other screenings [189, 198]. Sequence alignment of all putative RTA binding sites identified the consensus binding motif as TTCCAGGAT(N)(0-16)TTCCTGGGA [199]. Interestingly, most RTA binding sites identified in the Chip-on-Chip screening contain only half or part of this consensus sequence, suggesting the complexity of RTA transactivation mechanism. One simple explanation for these variations is that RTA binds to its targets through interactions with other viral or cellular regulatory proteins. Indeed, RTA transactivation of ORF-K8 and ORF57 depends on heterogeneous RREs, and the activation of ORF57 promoter requires an AP-1 binding site [192]. Similarly, autoactivation of RTA and RTA transactivation of viral lytic promoters such as MTA and thymidine kinase (TK, ORF21) promoters depend on Sp-1, octamer-binding protein-1 (Oct-1) and XBP-1 [158, 200–204]. Several studies have shown that RTA is recruited for RREs through interaction with RBP-J*κ* [205–207]. The RTA-RBP-J*κ* interaction converts the repressor into an activator, resulting in the activation of viral promoters. Consistent with these results, RBP-J*κ* is usually not detectable in KSHV-infected latent cells but is significantly upregulated during viral lytic replication, and intracellular-activated Notch1 is sufficient to reactivate KSHV from latency [208]. In addition, RTA stimulates DNA binding of RBP-J*κ* to activate the Notch pathway, which allows KSHV to complete the lytic cycle [209]. 

RTA transactivation function also depends on its interactions with other proteins. RTA activation of viral promoters depends on the recruitment of CBP, the SWI/SNF chromatin remodeling complex, and the TRAP/mediator coactivator into viral promoters [210]. The Brg1 subunit of SWI/SNF and the TRAP230 subunit of TRAP/mediator interact directly with RTA through a short acidic sequence in the carboxyl region. Genetic ablation of these interactions abolishes RTA activation of lytic genes and KSHV lytic replication. Additionally, RTA interacts with K-RBP (KSHV RTA binding protein), a homologue of the Kruppel-associated box-zinc finger proteins (KRAB-ZFPs), for its transactivation function [211–213]. The corepressor of K-RBP, Kruppel-associated box domain-associated protein-1 (KAP-1)/transcriptional intermediary factor 1beta, a cellular transcriptional repressor that controls chromosomal remodeling, also participates in the control of lytic replication [214]. During latency KAP-1 binds to viral lytic promoters to repress gene expression. Knockdown of KAP-1 is sufficient to induce KSHV reactivation. Both sumoylation and phosphorylation regulate KAP-1 association with heterochromatin. During reactivation, RAP-1 is phosphorylated at Ser 824, resulting in its decreased sumoylation and binding to the condense chromatin on viral promoters. Interestingly, RTA induction of viral lytic gene expression is also negatively correlated with reduced KAP-1 binding to viral promoters. These evidences point to an important role of KAP-1 for regulating KSHV latency.

The mechanism of RTA transactivation of K-bZIP is complex and is involved with an amplification process. K-bZIP physically interacts with and stabilizes the cellular transcription factor CCAAT/enhancer-binding protein-alpha (C/EBP*α*), leading to upregulated expression of C/EBP*α* and p21CIP1 proteins followed by G0/G1 cell cycle arrest [215, 216]. RTA interacts with C/EBP*α*, and both K-bZIP and RTA cooperate with C/EBP*α* to activate the K-bZIP promoter through binding to a proximal C/EBP*α* binding site that also serves as an RRE [215]. C/EBP*α* also activates the promoters of RTA, PAN, and MTA through direct binding of the C/EBP*α* and RTA complex [216, 217]. Therefore, besides direct DNA binding, RTA cooperates with different cellular transcriptional factors to upregulate various downstream viral genes.

Interestingly, RTA exhibits a ubiquitin E3 ligase activity and targets multiple cellular and viral proteins for proteasome-mediated degradation [218]. One of the RTA degradation targets is IRF-7, a key mediator of type I IFN induction [219]. Since IFN signaling plays an important role in suppressing viral lytic replication, this finding suggests that RTA might overcome the host innate immune defenses during KSHV reactivation. Another cellular protein targeted by RTA is Hey1, which interacts with repressor mSin3A. Hey1 degradation disrupts the interaction between Hey1 and mSin3A. Hey1 suppresses RTA expression by direct binding to the RTA promoter [220]. By targeting Hey1 for degradation, RTA upregulates its own expression. Together, these results suggest that RTA regulates viral lytic replication by promoting protein degradation of several cellular repressors.

Finally, RTA might contribute to KSHV lytic DNA replication [221]. The left OriLyt (OriLyt-L) lies between ORF-K4.2 and -K5 and is composed of a region encoding various transcription factor binding sites, an A+T-rich region, and a G+C repeat. The right OriLyt (OriLyt-R) is mapped between ORF69 and vFLIP and is an inverted duplication of OriLyt-L. Interestingly, each OriLyt contains an RRE [222]. It was shown that binding of RTA to this RRE is essential for OriLyt-dependent DNA replication [167, 222, 223]. The presence of this *cis*-element and a downstream TATA box suggests that this region serves as an RTA-dependent promoter, and a transcription event may be necessary for OriLyt-dependent DNA replication.

#### 3.3.2. MTA

MTA is required for KSHV lytic replication. Genetic knockout of MTA abolishes KSHV productive lytic replication [224, 225]. MTA protein possesses domains with putative transcriptional and posttranscriptional functions [226]. MTA itself does not enhance the expression of RTA or other lytic genes alone. Rather, it binds to proteins with transcriptional regulatory functions. For example, MTA synergizes with RTA to regulate expression of viral genes in a promoter- and cell line-specific manner [226, 227]. MTA and RTA directly interact with each other, and both proteins are found in RTA promoter during lytic replication. A DNA element conserved in multiple KSHV promoters is necessary but not sufficient for this synergy. A putative A/T hook domain within MTA mediates DNA binding and transcriptional initiation [226]. When RTA transactivation function is eliminated, MTA no longer affects the expression of viral genes suggesting that their synergistic effect depends on RTA's transactivation function. Thus, MTA facilitates the cascade of viral gene expression during lytic replication through physical interaction with RTA. 

MTA enhances the accumulation of specific viral and cellular mRNAs [228]. This effect is more efficient for intronless RNAs and is independent of transcriptional regulation [229, 230]. MTA shuttles between nuclei and cytoplasm, and promotes nuclear export of nonspliced RNAs [229, 231, 232]. Besides, MTA stabilizes RNAs and stimulates translation of mRNAs that contain internal ribosome entry sites [233].

#### 3.3.3. K-bZIP

K-bZIP physically interacts with RTA. This interaction involves K-bZIP's basic domain (aa122-189) and a specific RTA region (aa499-550) [234]. In contrast to MTA, K-bZIP represses RTA transactivation of MTA promoter in a dose-dependent manner [212, 213]. K-bZIP also represses the RTA autoactivation [234]. A K-bZIP mutant lacking the RTA interaction domain fails to repress RTA-mediated transactivation, suggesting the requirement of this interaction for K-bZIP's repression effect. Interestingly, such repression is not seen for the PAN promoter. Thus, K-bZIP repression of RTA-mediated transactivation is likely promoter dependent. 

K-bZIP is sumoylated at the lysine 158 residue and is associated with Ubc9 both in a cell-free system and in BCBL-1 cells [235]. K-bZIP sumoylation is required for transcription repression. Acting as a SUMO adaptor, K-bZIP recruits Ubc9 to specific viral promoters to exert its transcriptional repression activity [235]. Together, these results suggest that K-bZIP is involved in a feedback circuit to turn off its own expression and possibly other RTA-activated lytic genes. Nevertheless, a genome-wide analysis of K-bZIP's transcriptional effect on 83 putative KSHV gene promoters showed that 34 viral promoters were activated by RTA and 21 promoters by K-bZIP alone [236]. However, when RTA and K-bZIP were combined, 3 RTA-responsive promoters were repressed by K-bZIP. Therefore, K-bZIP might also transactivate some viral lytic genes during KSHV reactivation.

K-bZIP interacts with some other proteins such as a serine/threonine protein kinase (vPK) encoded by ORF36 to exert its regulatory effect [237]. During reactivation, vPK, K-bZIP, and a polymerase processivity factor are colocalized with the viral DNA replication/transcription compartments, and both K-bZIP and vPK are recruited to K-bZIP target promoters and OriLyt. vPK strongly phosphorylates K-bZIP at threonine 111 [238], resulting in a resistance of K-bZIP to sumoylation and a reduction of its transcription repression activity [238]. Thus, K-bZIP and vPK cooperate to modulate viral transcription and replication [237].

The fact that K-bZIP binds to OriLyts suggests a role for K-bZIP in lytic DNA replication [167]. LANA also binds to OriLyts and represses origin-dependent lytic DNA replication. However, this LANA repression effect can be overcome by K-bZIP [239]. Since LANA interacts with OriLyts in the absence of K-bZIP, its suppression of lytic DNA replication is likely mediated by direct DNA binding. In contrast, interaction of K-bZIP with OriLyts depends on LANA expression [239]. Therefore, origin-dependent DNA replication requires not only the core replication proteins but also both RTA and K-bZIP.

Collectively, these studies have shown that K-bZIP has dual functions in modulating KSHV life cycle: on one hand, it facilitates lytic DNA replication, on the other hand, it serves as a feedback modulator to repress lytic gene expression. These two functions might be independent of each other [240]. Consistent with these results, knock-down of K-bZIP in KSHV-infected cells BCBL-1 and BC-3 led to dramatic decreases in the expression of RTA, MTA and ORF26 transcripts, reduced RTA and ORF-K8.1 protein levels, and severely impaired viral DNA replication and virion production [241]. Thus, K-bZIP is essential for lytic gene expression, DNA replication, and virus production in PEL cells.

## 4. Conclusions

Remarkable advancements have been made in our understanding of the mechanisms of KSHV latency and lytic replication in the last several years (Figure 1). Given the critical roles of both replication modes in viral life cycle and pathogenesis, these progresses have significantly advanced the biology of KSHV and should provide the scientific basis for developing novel intervention approaches to inhibit KSHV infection and KSHV-induced malignancies. With the rapid advancement of novel technologies such as high-throughput sequencing and real-time high-resolution imaging, it is expected that more comprehensive understanding of the molecular basis of KSHV life cycle will be made in the next few years.
